# Outpatient Treatment with Isavuconazole for Recurrent Chronic Cavitary Pulmonary Aspergillosis in a Residual Post-Lobectomy Cavity: A Case Report

**DOI:** 10.3390/idr18040091

**Published:** 2026-08-20

**Authors:** Marialuisa Valente, Giovanni Fumagalli, Valentina Caputo, Niccolò Riccardi, Lucia Allavena, Maurizio Ferrarese, Luigi Ruffo Codecasa

**Affiliations:** 1 Department of Medical and Surgical Sciences, University of Modena and Reggio Emilia, 41121 Modena, Italy; 2 Regional TB Reference Centre and Laboratory, Villa Marelli Institute, ASST Grande Ospedale Metropolitano Niguarda, 20149 Milan, Italy; 3 Department of Surgical Pathology, ASST Grande Ospedale Metropolitano Niguarda, 20162 Milan, Italy

**Keywords:** chronic pulmonary aspergillosis (CPA), chronic cavitary pulmonary aspergillosis (CCPA), Aspergillus fumigatus, isavuconazole, pulmonary infection, post-surgical cavity

## Abstract

Background: Chronic pulmonary aspergillosis (CPA) is a slowly progressive pulmonary fungal disease, predominantly caused by *Aspergillus fumigatus*, which develops in patients with pre-existing structural lung abnormalities. Chronic cavitary pulmonary aspergillosis (CCPA), the most common form of CPA, is characterized by one or more pulmonary cavities, with or without intracavitary fungal balls (aspergillomas), and requires prolonged oral triazole therapy. Long-term treatment may be limited by adverse events, drug–drug interactions, and the need for therapeutic drug monitoring with conventional azoles. Case Presentation: A 69-year-old former smoker with a history of breast cancer and right upper lobectomy for pulmonary adenocarcinoma presented with fever, productive cough, hyporexia, sarcopenia, and an unintentional 6 kg weight loss over three months. Two years earlier, she had undergone atypical pulmonary resection for an *Aspergillus fumigatus* aspergilloma and had been treated with amphotericin B followed by voriconazole for six months. HRCT revealed a recurrent intracavitary fungal lesion within a residual post-surgical cavity with extensive inflammatory consolidation. Bronchoalveolar lavage demonstrated a positive galactomannan index (4.74) and septate fungal hyphae on cytology, supporting the diagnosis of recurrent CCPA. Treatment with oral isavuconazole resulted in clinical recovery, weight gain, and complete radiological resolution after six months, without treatment-related adverse events or QTc abnormalities. Conclusions: This case highlights the successful outpatient management of CCPA developing within a persistent post-lobectomy cavity despite previous surgical resection and antifungal therapy. The favourable clinical response, excellent tolerability, and absence of treatment-related toxicity support the growing evidence that isavuconazole may represent a valuable therapeutic option for selected patients with recurrent CCPA.

## 1. Background

*Aspergillus* spp. are ubiquitous environmental fungi that can cause a broad spectrum of pulmonary diseases, ranging from colonization and allergic bronchopulmonary aspergillosis (ABPA) to chronic pulmonary aspergillosis (CPA) and invasive pulmonary aspergillosis (IPA) [1]. The clinical phenotype is largely determined by the interaction between host immune status and underlying structural lung abnormalities [1]. While IPA predominantly affects severely immunocompromised individuals with risk factors such as prolonged neutropenia or hematological malignancies and may disseminate beyond the lungs mainly to the central nervous system and the liver, CPA usually develops in immunocompetent or mildly immunocompromised patients with pre-existing structural lung disease and typically remains confined to the respiratory tract [1,2].

CPA is a slowly progressive pulmonary fungal disease caused by *Aspergillus* spp. It is estimated to affect approximately 240,000 people in Europe and nearly three million individuals worldwide, representing an important cause of chronic respiratory morbidity and mortality [3].

CPA comprises a spectrum of clinical entities, including simple aspergilloma, chronic cavitary pulmonary aspergillosis (CCPA), chronic fibrosing pulmonary aspergillosis (CFPA), *Aspergillus* nodules, and subacute invasive pulmonary aspergillosis (SAIA) [1]. Among these, CCPA is the most common form and is characterized by one or more pulmonary cavities, with or without intracavitary fungal balls (aspergillomas), associated with microbiological or serological evidence of *Aspergillus* infection and radiological progression over at least three months [2].

Previous pulmonary tuberculosis remains the leading predisposing condition worldwide; however, chronic obstructive pulmonary disease, fibrotic interstitial lung diseases, sarcoidosis, emphysema, and residual post-operative cavities are increasingly recognized as important risk factors in high-income countries [2,4].

High-resolution computed tomography (HRCT) is the imaging modality of choice, while microbiological confirmation may rely on a combination of fungal culture, histopathology, galactomannan detection on bronchoalveolar lavage (BAL), and *Aspergillus*-specific IgG antibodies [2,5].

Long-term oral triazole therapy represents the cornerstone of treatment, whereas surgery is generally reserved for selected patients with localized disease or uncontrolled hemoptysis [2]. Herein, we report a case of recurrent CCPA arising within a residual post-lobectomy cavity that was successfully managed with prolonged outpatient isavuconazole therapy.

## 2. Case Presentation

**Figure 1:**
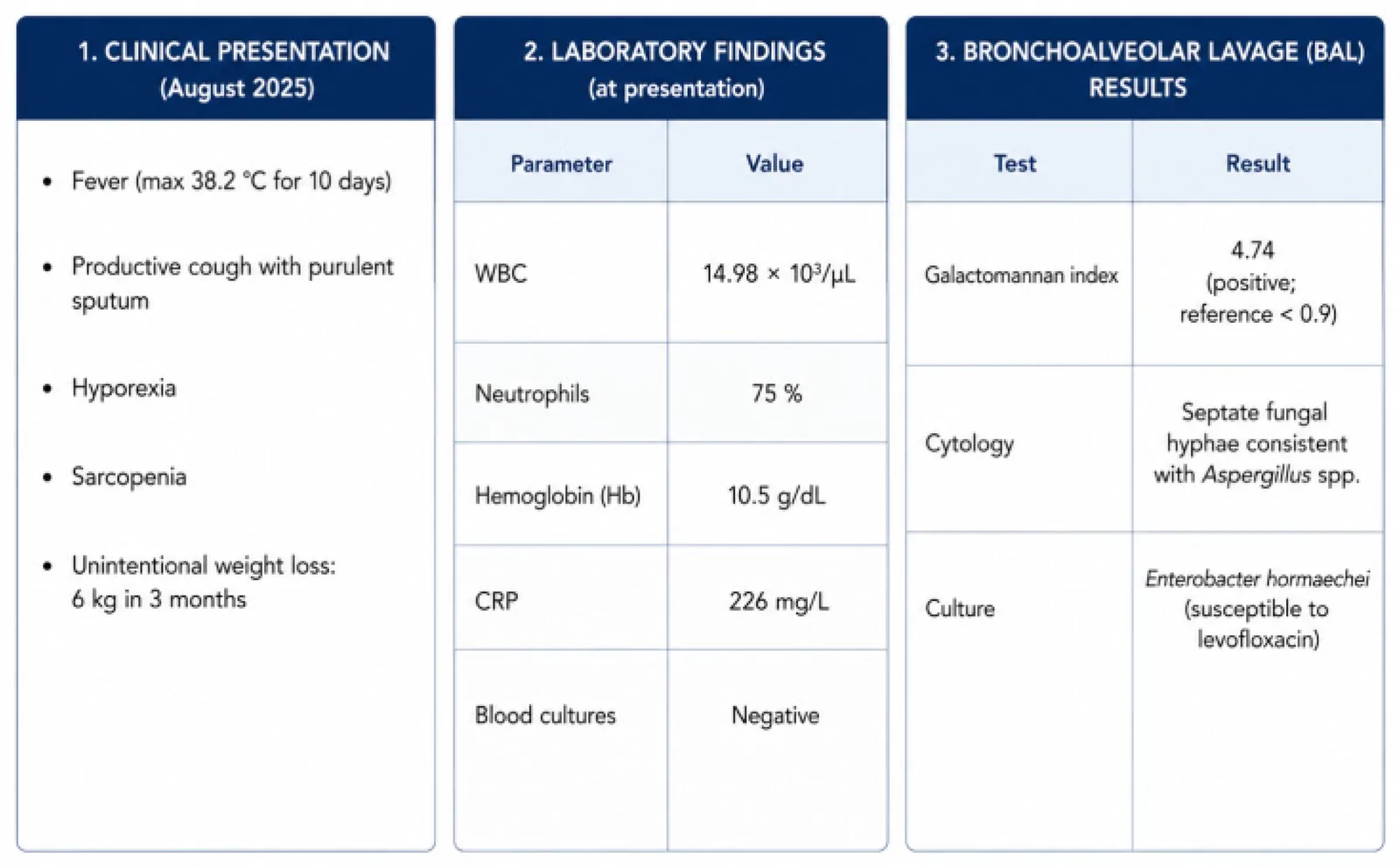
Clinical and laboratory findings.

A 69-year-old former smoker presented to the Emergency Department in August 2025 with a 10-day history of fever (maximum temperature 38.2 °C), productive cough with purulent sputum, hyporexia, and an unintentional weight loss of 6 kg over the preceding three months [Figure 1].

Her past medical history was notable for invasive ductal breast carcinoma treated with surgery followed by adjuvant chemotherapy and radiotherapy. In 2015, she underwent right upper lobectomy with hilar-mediastinal lymphadenectomy for pulmonary adenocarcinoma.

During routine radiological follow-up in 2023, chest CT revealed a cavitary lesion involving the apical segment of the right lower lobe containing an intracavitary vegetating mass suggestive of aspergilloma. The patient subsequently underwent atypical pulmonary resection. Histopathological and microbiological examination of the surgical specimen identified *Aspergillus fumigatus*, susceptible to amphotericin B and voriconazole. She was treated with amphotericin B followed by oral voriconazole for a total of six months, with complete clinical recovery.

**Figure 2:**
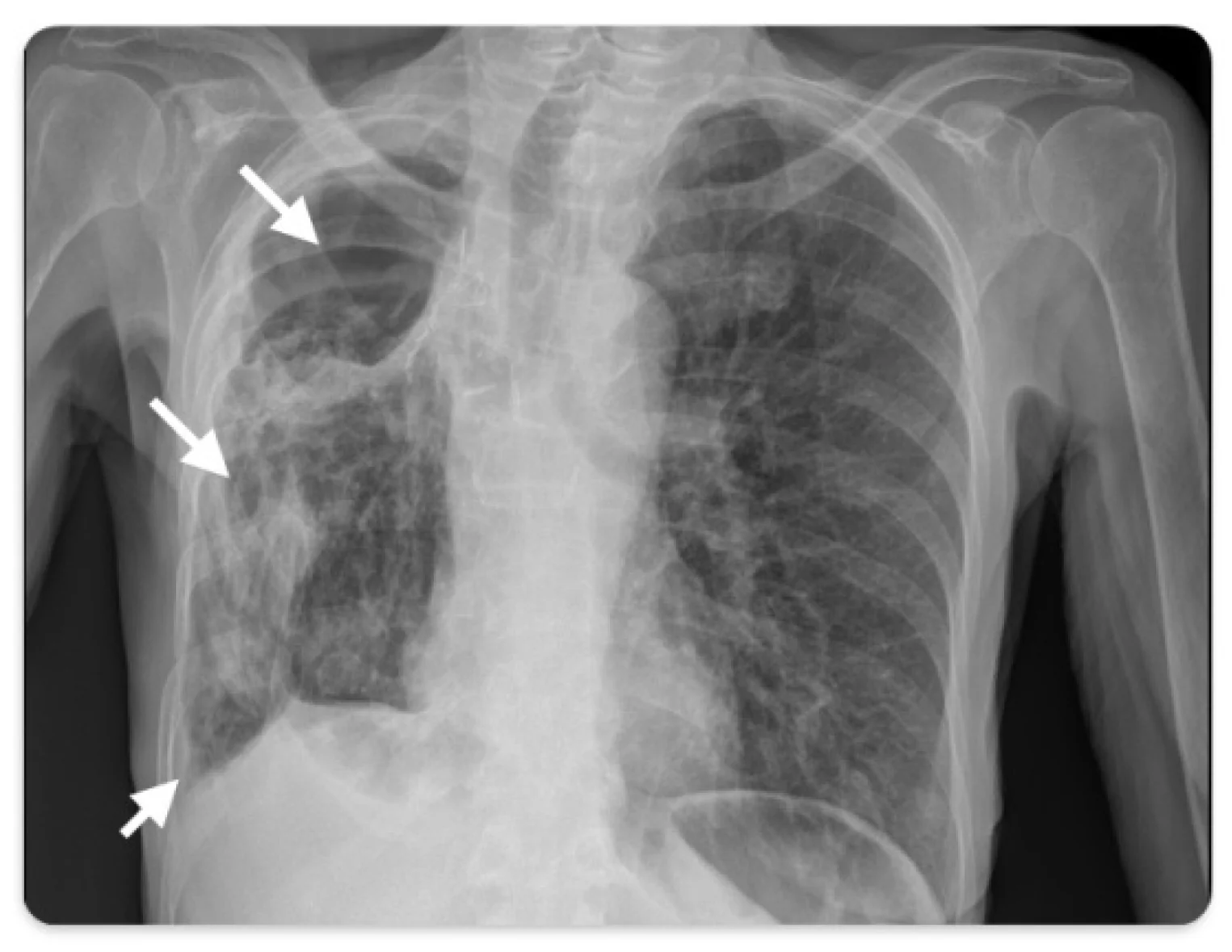
Chest X-ray showing hyperlucency of the right upper lung. Extensive consolidation involving the right middle and lower lung, as well as minimal right pleural effusion (see white arrows in the image).

At presentation to the Emergency Department, laboratory investigations demonstrated leukocytosis (14.98 × 10^3^/μL) and markedly elevated C-reactive protein levels (226 mg/L) [Figure 1]. Chest radiography showed volume loss of the right lung related to the previous upper lobectomy, a large hyperlucent area in the upper right hemithorax corresponding to the known post-surgical cavity, extensive consolidation involving the right middle and lower lung fields, and a small right pleural effusion [Figure 2].

**Figure 3:**
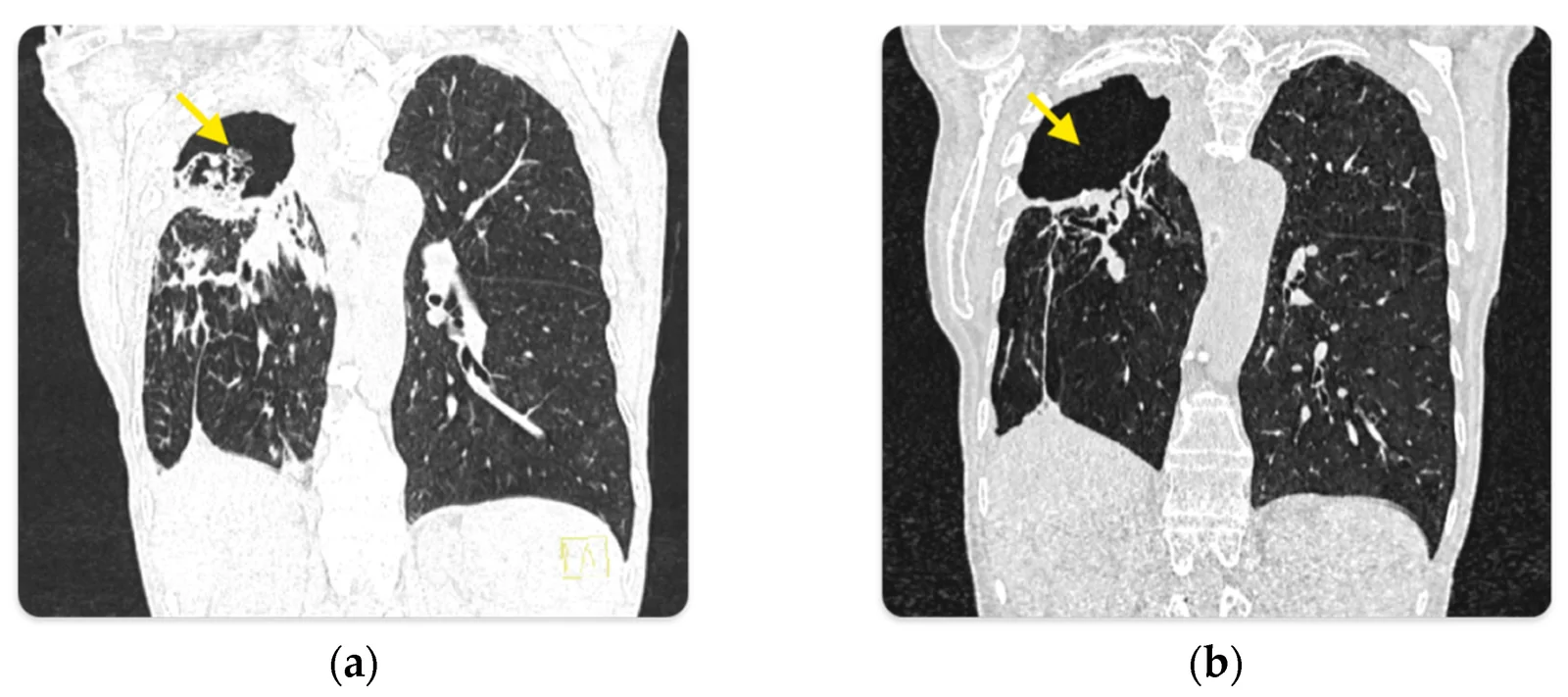
The figure shows the CT scans performed respectively before and after the Isavuconazole treatment: (**a**) coronal chest CT scan showing extensive air component within the apical right pleural cavity, with an irregular serpiginous structure inside typical of chronic cavitary pulmonary aspergillosis (see yellow arrow in the image); (**b**) chest HRTC after six months of treatment showing the absence of endocavitary neoformation at the upper right lobe and the almost complete resolution of the underlying consolidation (see yellow arrow in the image).

HRCT demonstrated a large residual right apical pleural cavity containing an irregular serpiginous intracavitary lesion highly suggestive of fungal colonization [Figure 3a]. Extensive heterogeneous consolidation of the adjacent lung parenchyma, mucus impaction of the peripheral bronchi of the right lower lobe with multiple peribronchial inflammatory consolidations, a 6 mm pseudonodular opacity in the left upper lobe, and a small loculated right pleural effusion were also observed.

**Figure 4:**
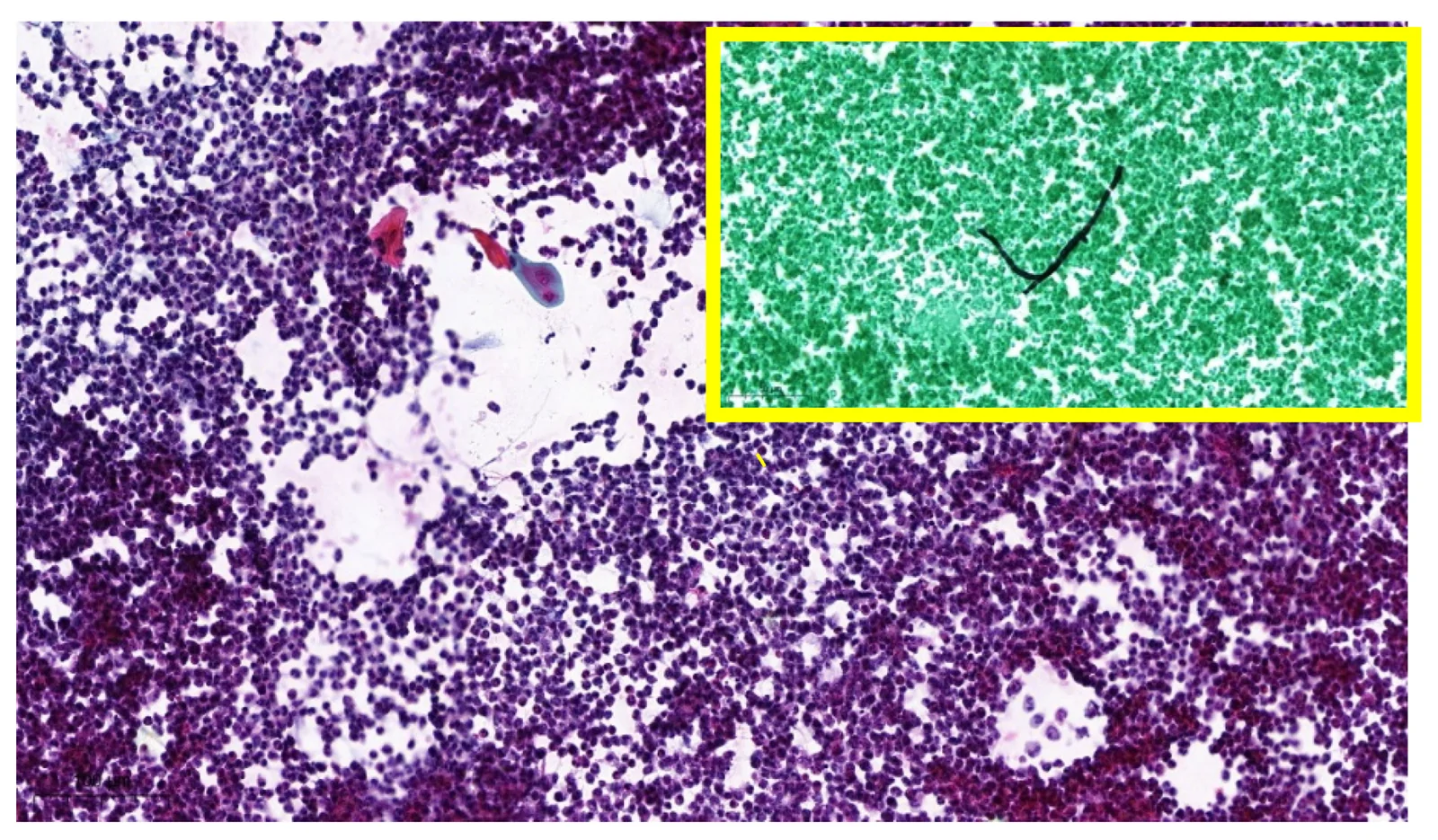
Cytological examination of bronchoalveolar lavage demonstrated, on a background rich in neutrophilic and eosinophilic granulocytes, the presence of hyphae, morphologically referable to *Aspergillus*, positive to Grocott staining.

The patient was admitted to the Infectious Diseases Unit for further diagnostic work-up. Flexible bronchoscopy with BAL was performed. BAL culture yielded *Enterobacter hormaechei* (>10^6^ CFU/mL), whereas no fungal pathogen was isolated. BAL galactomannan was strongly positive (index 4.74), cytological examination demonstrated septate fungal hyphae morphologically consistent with *Aspergillus* species [Figure 4], and serum galactomannan and (1→3)-β-D-glucan assays were negative. *Aspergillus*-specific IgG testing was not available during this clinical episode. Blood cultures obtained because of transient post-bronchoscopy fever remained negative.

Following hospital discharge, the patient was referred to our Regional tuberculosis and NTM reference centre for further evaluation and management. Based on the previous history of pulmonary aspergillosis together with the clinical presentation, radiological findings, and microbiological evidence, a diagnosis of recurrent chronic CCPA was established. Oral isavuconazole was initiated using the standard loading regimen (200 mg every 8 h for the first 48 h), followed by maintenance therapy at 200 mg once daily.

After one month of treatment, the patient reported complete resolution of fever and marked improvement in respiratory symptoms. Serial chest radiographs obtained during outpatient follow-up documented progressive radiological improvement.

After approximately six months of therapy, the patient had regained 5 kg (45 kg versus 40 kg at diagnosis), and repeat HRCT showed complete radiological resolution of the intracavitary fungal lesion, with near-complete resolution of the surrounding pulmonary consolidations [Figure 3b]. Throughout treatment, serial electrocardiograms showed normal QTc intervals, and no hepatotoxicity or other treatment-related adverse events were observed. At the time of writing, the patient has completed eight months of isavuconazole therapy and remains clinically stable while awaiting further radiological reassessment with an expected treatment duration of twelve months.

## 3. Discussion

CCPA is the most frequent clinical manifestation of CPA and typically develops in patients with pre-existing structural lung abnormalities that facilitate persistent Aspergillus colonization and intracavitary fungal growth. While pulmonary tuberculosis remains the leading predisposing condition worldwide, the epidemiology of CPA is evolving in high-income countries, where chronic obstructive pulmonary disease, emphysema, fibrotic interstitial lung diseases, and residual postoperative cavities are increasingly recognized as important risk factors. In contrast to invasive pulmonary aspergillosis (IPA), which is characterized by angioinvasion and predominantly occurs in severely immunocompromised patients, CCPA generally arises in patients with underlying structural lung disease and limited or no systemic immunosuppression. Moreover, the clinical course of CCPA is typically chronic and progressive over months or years, rather than the rapidly progressive presentation characteristic of IPA. [2,4,6]. Structural distortion of the lung, impaired mucociliary clearance, and the persistence of poorly vascularized cavities represent a favourable anatomical substrate for persistent *Aspergillus* colonization, explaining why recurrence may occur even after apparently successful surgery and previous antifungal therapy [2,5].

Our patient represents a paradigmatic example of recurrent CCPA developing within a residual post-lobectomy cavity. Although surgical resection is considered curative for selected patients with simple aspergilloma, recurrence remains possible when structural abnormalities persist, emphasizing the importance of long-term clinical and radiological surveillance in patients with permanent cavitary lesions [2,7,8].

The diagnosis of recurrent CPA remains challenging because constitutional symptoms, cough, and weight loss are non-specific and frequently mimic bacterial pneumonia, lung cancer recurrence, mycobacterial disease or progression of underlying chronic lung disease [2,5].

*Aspergillus*-specific IgG is considered the preferred serological test for chronic pulmonary aspergillosis; however, no single microbiological or immunological test is sufficient to establish or exclude the diagnosis. Instead, the diagnosis relies on the integration of compatible clinical presentation, characteristic radiological findings, microbiological and/or immunological evidence, and exclusion of alternative diagnoses [2]. Negative fungal cultures and negative serum fungal biomarkers do not exclude CPA, given the limited sensitivity of these tests in chronic, non-angioinvasive disease [1,2]. In our patient, the diagnosis was supported by the previous microbiological identification of *Aspergillus fumigatus*, characteristic HRCT findings, a strongly positive BAL galactomannan, cytological evidence of septate fungal hyphae, and validated by the subsequent favourable clinical and radiological response to isavuconazole.

The optimal duration of antifungal treatment remains uncertain and should be individualized according to clinical and radiological response, inflammatory and serological markers. [9]. Although current guidelines recommend treatment for at least six months, recent evidence, including a randomized clinical trial, suggests that prolonged therapy, often extending beyond 12 months, may reduce relapse rates in patients with persistent cavitary disease [2,7,8,10]. Koyama and colleagues demonstrated that recurrence after discontinuation of antifungal therapy occurs predominantly in patients with residual cavities, highlighting the need for individualized treatment duration and prolonged post-treatment follow-up [8].

Itraconazole and voriconazole remain the recommended first-line oral antifungal agents; however, their long-term use is frequently limited by hepatotoxicity, neurotoxicity, clinically relevant CYP450-mediated drug interactions, and marked interindividual pharmacokinetic variability, making therapeutic drug monitoring (TDM) routinely recommended [2,11]. These limitations become particularly relevant in elderly patients and in those with polypharmacy.

Isavuconazole has emerged as a promising alternative because of its predictable pharmacokinetic profile, excellent oral bioavailability, good tolerability, and lower potential for drug–drug interactions [12,13,14]. Unlike itraconazole and voriconazole, routine TDM is generally not recommended owing to lower interindividual pharmacokinetic variability, although drug concentration monitoring may still be considered in selected clinical scenarios, including suspected malabsorption, unexpected therapeutic failure, or extreme body weight [11,12,13]. Another distinctive pharmacological characteristic is its tendency to shorten rather than prolong the QT interval, which may represent an additional advantage in elderly patients with cardiovascular comorbidities or polypharmacy [12,13].

Although evidence supporting isavuconazole in CPA remains limited compared with invasive aspergillosis, the available literature is steadily expanding. A recent systematic review identified 15 published studies evaluating isavuconazole in CPA, including retrospective cohorts, comparative studies, and case reports, reporting clinical efficacy comparable to voriconazole together with improved tolerability and treatment persistence [14]. Likewise, Morante Tinoco et al. [15] reported a case of CCPA in a patient with a similar post-surgical cavity which was successfully managed with oral antifungal therapy. Similarly, Bongomin and colleagues observed significantly fewer adverse drug reactions in patients treated with isavuconazole than in those receiving voriconazole (60% vs. 86%; *p* = 0.02), while clinical outcomes remained comparable [16].

In our case report, prolonged outpatient treatment with isavuconazole resulted in complete clinical recovery, weight gain, progressive radiological resolution of pulmonary consolidations, and complete disappearance of the intracavitary fungal lesion without hepatotoxicity, QTc abnormalities, or treatment discontinuation during the first eight months of treatment. Although no definitive conclusions can be drawn from a single case, our experience adds to the growing body of evidence supporting isavuconazole as a safe, effective, and well-tolerated alternative for the management of recurrent CCPA [14,16].

## 4. Conclusions

This case underscores the importance of long-term clinical and radiological surveillance in patients with persistent structural lung abnormalities even after successful surgery and antifungal therapy for CCPA. Furthermore, it illustrates that prolonged outpatient treatment with isavuconazole can achieve clinical and radiological remission with excellent tolerability in selected patients with recurrent disease. Although prospective studies are needed to better define the role of isavuconazole in CPA, our findings further support the growing evidence for its use as a valuable therapeutic option.

## Data Availability

No additional clinical data are available.
